# STAT2 dependent Type I Interferon response promotes dysbiosis and luminal expansion of the enteric pathogen *Salmonella* Typhimurium

**DOI:** 10.1371/journal.ppat.1007745

**Published:** 2019-04-22

**Authors:** R. Paul Wilson, Sarah A. Tursi, Glenn J. Rapsinski, Nicole J. Medeiros, Long S. Le, Kevin P. Kotredes, Sajan Patel, Elisabetta Liverani, Shuang Sun, Wenhan Zhu, Laurie Kilpatrick, Sebastian E. Winter, Ana M. Gamero, Çagla Tükel

**Affiliations:** 1 Department of Microbiology and Immunology, Lewis Katz School of Medicine, Temple University, Philadelphia, PA, United States of America; 2 Department of Medical Genetics and Molecular Biochemistry, Lewis Katz School of Medicine, Temple University, Philadelphia, PA, United States of America; 3 Department of Thoracic Medicine and Surgery, Lewis Katz School of Medicine, Temple University, Philadelphia, PA, United States of America; 4 Department of Microbiology, University of Texas Southwestern Medical Center Dallas, TX, United States of America; University of California, Davis, UNITED STATES

## Abstract

The mechanisms by which the gut luminal environment is disturbed by the immune system to foster pathogenic bacterial growth and survival remain incompletely understood. Here, we show that STAT2 dependent type I IFN signaling contributes to the inflammatory environment by disrupting hypoxia enabling the pathogenic *S*. Typhimurium to outgrow the microbiota. *Stat2*^*-/-*^ mice infected with *S*. Typhimurium exhibited impaired type I IFN induced transcriptional responses in cecal tissue and reduced bacterial burden in the intestinal lumen compared to infected wild-type mice. Although inflammatory pathology was similar between wild-type and *Stat2*^*-/-*^ mice, we observed decreased hypoxia in the gut tissue of *Stat2*^*-/-*^ mice. Neutrophil numbers were similar in wild-type and *Stat2*^*-/-*^ mice, yet *Stat2*^*-/-*^ mice showed reduced levels of myeloperoxidase activity. *In vitro*, the neutrophils from *Stat2*^*-/-*^ mice produced lower levels of superoxide anion upon stimulation with the bacterial ligand *N*-formylmethionyl-leucyl-phenylalanine (fMLP) in the presence of IFNα compared to neutrophils from wild-type mice, indicating that the neutrophils were less functional in *Stat2*^*-/-*^ mice. Cytochrome *bd-*II oxidase-mediated respiration enhances *S*. Typhimurium fitness in wild-type mice, while in *Stat2*^*-/-*^ deficiency, this respiratory pathway did not provide a fitness advantage. Furthermore, luminal expansion of *S*. Typhimurium in wild-type mice was blunted in *Stat2*^*-/-*^ mice. Compared to wild-type mice which exhibited a significant perturbation in Bacteroidetes abundance, *Stat2*^*-/-*^ mice exhibited significantly less perturbation and higher levels of Bacteroidetes upon *S*. Typhimurium infection. Our results highlight STAT2 dependent type I IFN mediated inflammation in the gut as a novel mechanism promoting luminal expansion of *S*. Typhimurium.

## Author summary

The spread of invading microbes is frequently contained by an inflammatory response. Yet, some pathogenic microbes have evolved to utilize inflammation for niche generation and to support their metabolism. Here, we demonstrate that *S*. Typhimurium exploits type I IFN signaling, a prototypical anti-viral response, to foster its own growth in the inflamed gut. In the absence of STAT2-dependent type I IFN, production of neutrophil reactive oxygen species was impaired, and epithelial metabolism returned to homeostatic hypoxia. Consequently, *S*. Typhimurium was unable to respire in the absence of type I IFN, and failed to expand in the gut lumen. Furthermore, perturbation of the gut microbiota was dependent on type I IFN signaling. Taken together, our work suggests that *S*. Typhimurium utilizes STAT2-dependent type I IFN signaling to generate a niche in the inflamed intestinal tract and outcompete the gut microbiota.

## Introduction

A healthy gastrointestinal microbiota is characterized by the dominance of obligate anaerobic members of the phyla Bacteroidetes and Firmicutes. The expansion of facultative anaerobic *Enterobacteriaceae* (phylum Proteobacteria) is considered a microbial signature for gut inflammation and dysbiosis [1, 2]. This signature is observed in severe human intestinal diseases including inflammatory bowel disease (IBD), [3–5] colorectal cancer [6] and necrotizing enterocolitis [7]. Several mechanisms by which the enteric pathogen, *Salmonella enterica* serovar Typhimurium, capitalizes on multiple processes induced by inflammation and outcompete the commensal have been described. Infection with *S*. Typhimurium starts with the invasion of intestinal epithelial cells using its type III secretion system (T3SS-1) [8]. After crossing the intestinal barrier, the bacterium is rapidly recognized by Pattern Recognition Receptors (PRRs), such as Toll-like receptors (TLRs) and Nod-like receptors (NLRs), and is internalized by macrophages or dendritic cells. In macrophages, *S*. Typhimurium survives using its T3SS-2 [9]. Epithelial invasion, recognition of Pathogen-Associated Molecular Patterns (PAMPs) and macrophage survival leads to the production of chemokines and cytokines triggering an inflammatory environment and acute colitis [10–12]. In the lumen, *S*. Typhimurium employs mechanisms to utilize unique respiratory electron acceptors (e.g. tetrathionate and nitrate) which are generated as byproducts of the inflammatory burst. Most commensal members of the microbiota are unable to metabolize nitrate and tetrathionate [13, 14]. As a result, *S*. Typhimurium outcompetes the healthy microbiota enabling its luminal expansion and eventually facilitating the transmission to subsequent hosts [13–16].

Although *S*. Typhimurium succeeds in expanding its luminal population during inflammation leading to a decline in the commensal microbiota, the coordinated actions of multiple immune cell defense pathways mediate the clearance of the pathogen. Activation of the Interferon (IFN) signaling pathway is critical for successful host defense against many infections. Type II IFN (IFN γ) plays a central role in generating inflammatory responses to clear *S*. Typhimurium [17–20]. However, the role of a closely related pathway involving the actions of type I IFNs (IFNs α and β) is less clear. Type I IFN signaling is well-documented as essential for mounting antiviral responses. Pre-exposure of cells to type I IFNs induces an antiviral state by blocking viral replication [21, 22]. It has recently become evident that activation of this pathway also plays a pivotal role during bacterial infections by acting directly or indirectly on many immune cell types including NK cells, T cells, B cells, Dendritic Cells (DCs), neutrophils and phagocytic cells. Depending on the bacterial agent, the role of type I IFNs exert seemingly opposing roles. For instance, while type I IFNs restrict the growth of *Legionella pneumophila* or *Streptococcal* species [23–27], activation of the same pathway impairs the clearance of intracellular *Mycobacterium tuberculosis* leading to tuberculosis [28, 29]. Recent studies highlighted the role of type I IFN signaling during systemic infection with *S*. Typhimurium. Mice deficient in type I IFN receptor (IFNAR), or IFN β exhibit greater resistance to *S*. Typhimurium [30]. Furthermore, type I IFNs are critical for inflammasome formation, caspase activation, and inflammatory cell death following infection with *S*. Typhimurium [31–34]. The role of this pathway during intestinal bacterial induced inflammation and the subsequent impact on the luminal bacterial population remains unclear.

**Fig 1:**
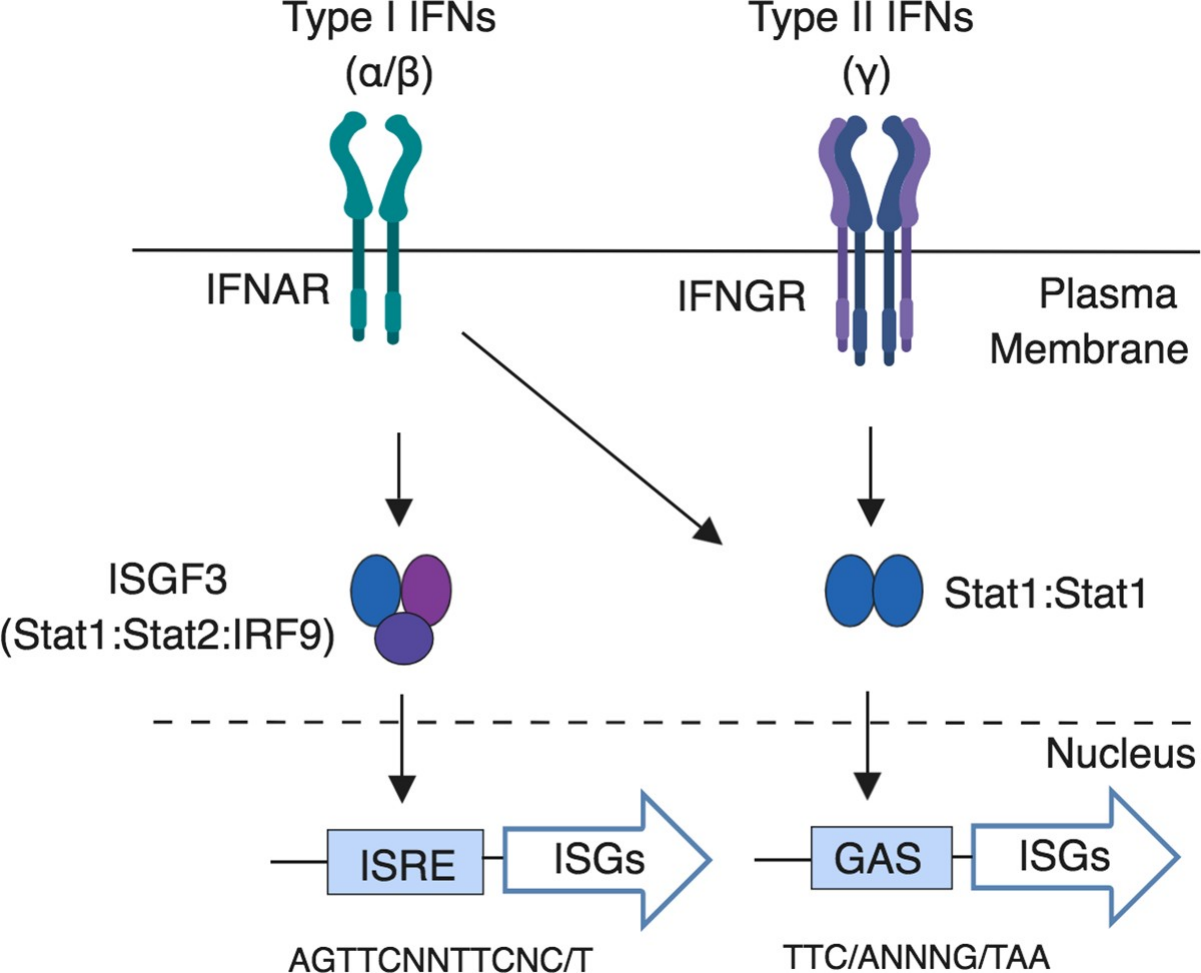
Activation of the type I IFN receptor (IFNAR) leads to the formation of the interferon stimulated gene factor 3 complex ISGF3 (STAT1:STAT2:IRF9 complex) which binds to the IFN stimulated response element (ISRE) promoter sequence leading to the activation of various interferon stimulated genes (ISGs) such as *isg15*, *irf7* and *cxcl10*. Both IFNAR and IFNGR activation leads to the formation of STAT1 homodimers that bind to GAS sequence leading to the production of additional ISGs.

IFNAR activation by type I IFNs (IFNs α and β) not only leads to the transcription of type I IFN stimulated genes (ISGs) induced by ISGF3, the heterotrimeric transcriptional complex composed of STAT1/STAT2/IRF9, but also by inflammatory gene activation via the formation of STAT1 homodimers. As STAT1 homodimers can also be activated by IFN γ, earlier studies that used *Ifnar*^-/-^ or *Stat1*^-/-^ mice did not clearly differentiate the contribution of each IFN pathway to driving inflammation (**Fig 1**). Here we used *Stat2*^*-/-*^ mice, which causes the genetic ablation of type I IFN signaling, in combination with the streptomycin pretreated mouse model to pinpoint the role of type I IFNs in host response to *Salmonella* infection. Overall, we conclude that STAT2-driven type I IFN response leads to the transmigration of functional neutrophils into the lumen creating a microaerophilic environment, which enables the pathogen to outgrow the microbiota.

## Results

### *STAT2*^*-/-*^ mice are more resistant to *S*. Typhimurium induced gastroenteritis

**Fig 2:**
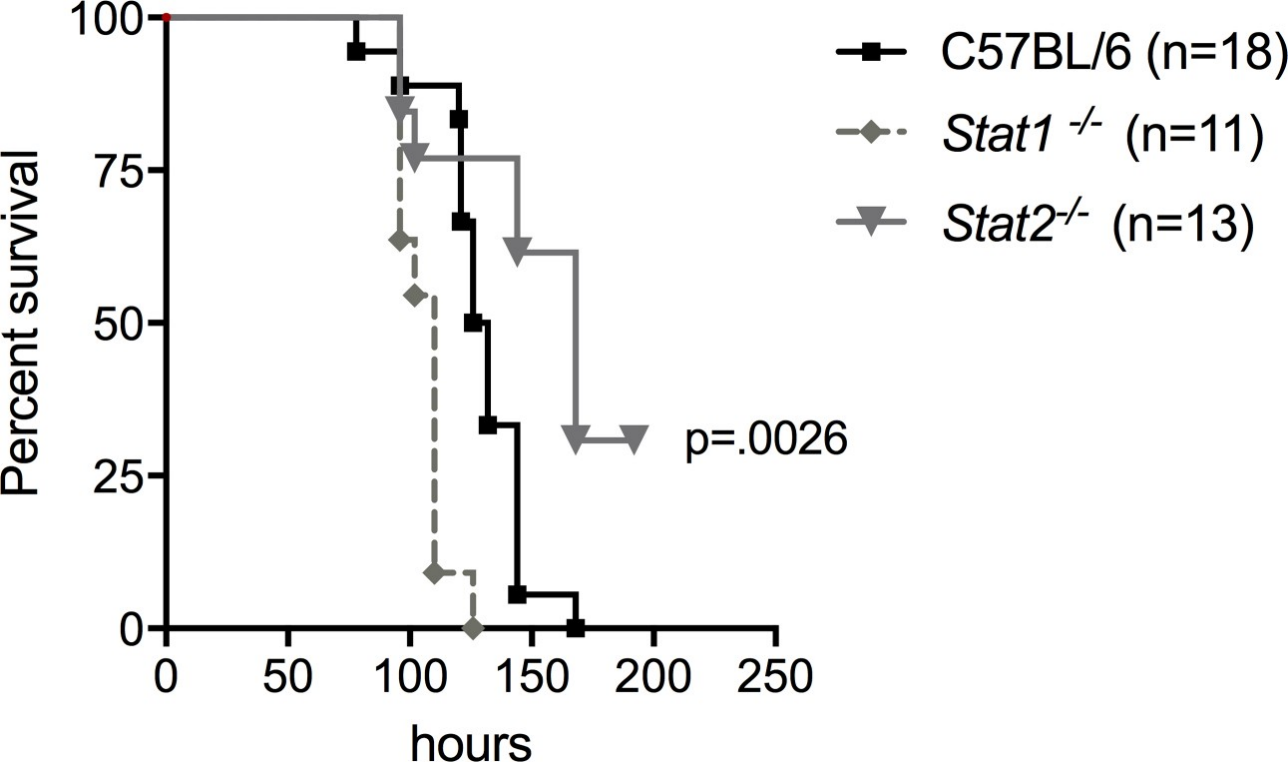
Survival of C57BL/6, *Stat2^-/-^* and *Stat1^-/-^* mice after oral *S.* Typhimurium infection. Percent survival of C57BL/6 (n = 18) black line, *Stat1^-/-^* (n = 11) red line, and *Stat2^-/-^* (n = 13) blue line mice for 8 days post oral 109 *Salmonella* Typhimurium infection following streptomycin pretreatment. The results from three independent experiments with at least 3 mice in each group were combined. *Stat2^-/-^* infected mice survived significantly longer, p = 0.0026.

Type I IFNs released during bacterial infections may affect many arms of the immune response including inhibition of bacterial invasion, amplification of the immune response and production of antimicrobial genes. To investigate the possible role of a STAT2-dependent type I IFN signaling pathway during *S*. Typhimurium induced intestinal infection, wild-type C57BL/6, *Stat1*^*-/-*^ (deficient in both IFN-α/β and IFN-γ signaling), and *Stat2*^*-/-*^ (deficient only in IFN-α/β signaling) mice were orally infected with 10^9^ CFU of *S*. Typhimurium following streptomycin pretreatment. All strains eventually succumbed to *S*. Typhimurium infection, but *Stat2*^*-/-*^ mice survived significantly longer than wild-type and *Stat1*^*-/-*^ mice (*p* = 0.0026) (**Fig 2**). This finding is notable because our previous study [35] reported increased mortality with *Stat2*^-/-^ mice during LPS-induced sepsis suggesting that type I IFNs play different roles when compared between mucosal and systemic sites during infection.

### STAT2 deficiency leads to altered intestinal immune responses during *S*. Typhimurium infection

**Fig 3:**
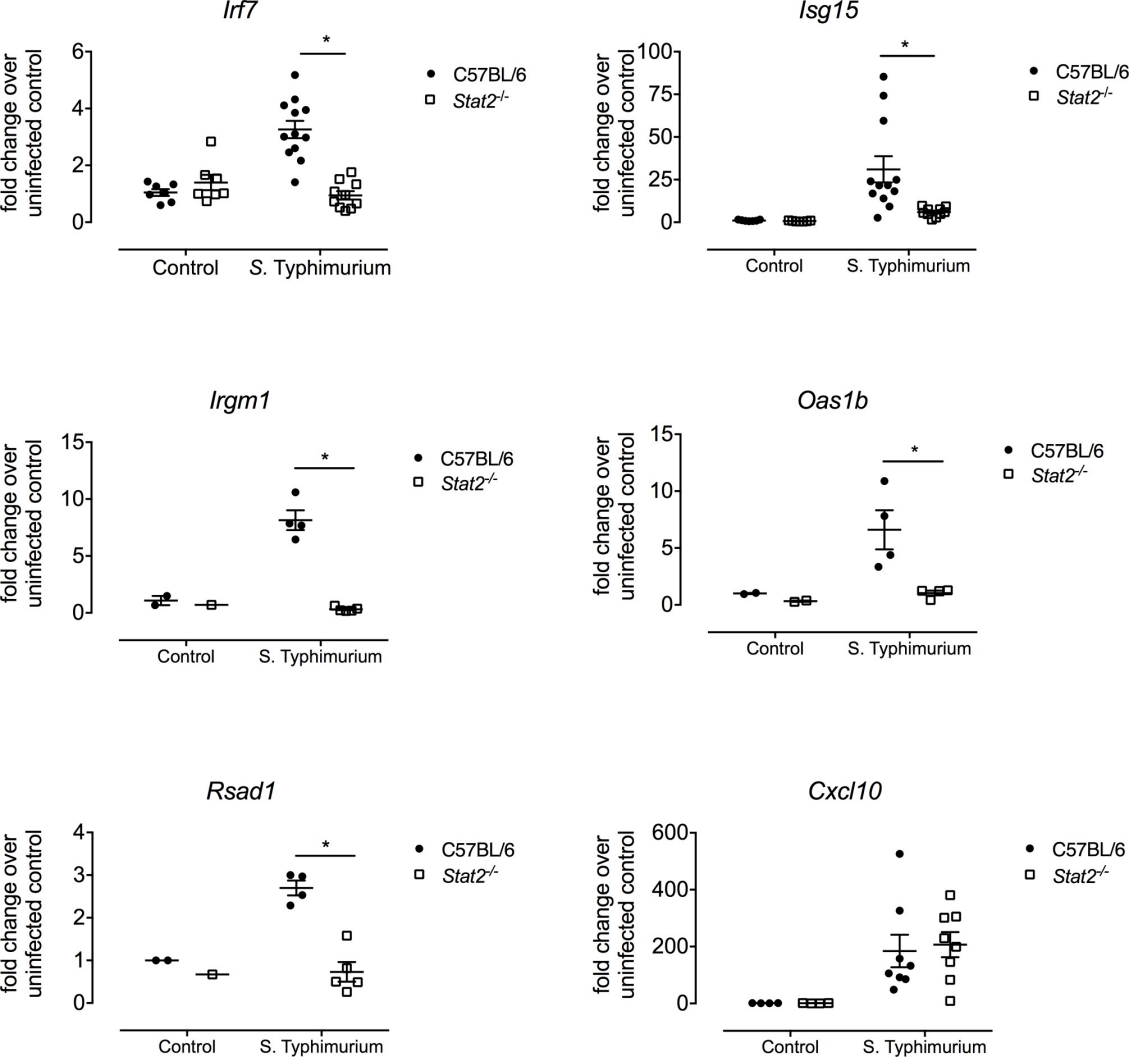
Transcript levels of various type I IFN stimulated genes in ceca of C57BL/6 and *Stat2^-/-^* mice 48 hours after *S.* Typhimurium infection. C57BL/6 and *Stat2^-/-^* mice were orally infected with 10^9^ STM following streptomycin pretreatment. Transcript levels of *Irf7, Isg15, Irgm1, Oas1b, Rsad1* and *Cxcl10* were determined by qPCR in the cecum of infected mice 48 post infection. Data was normalized to uninfected mice from each group. Each data point represents one analyzed mouse sample. Mean and SE were calculated by averaging results from three independent experiments. *p <0.05 as determined by Students t-test.

**Fig 4:**
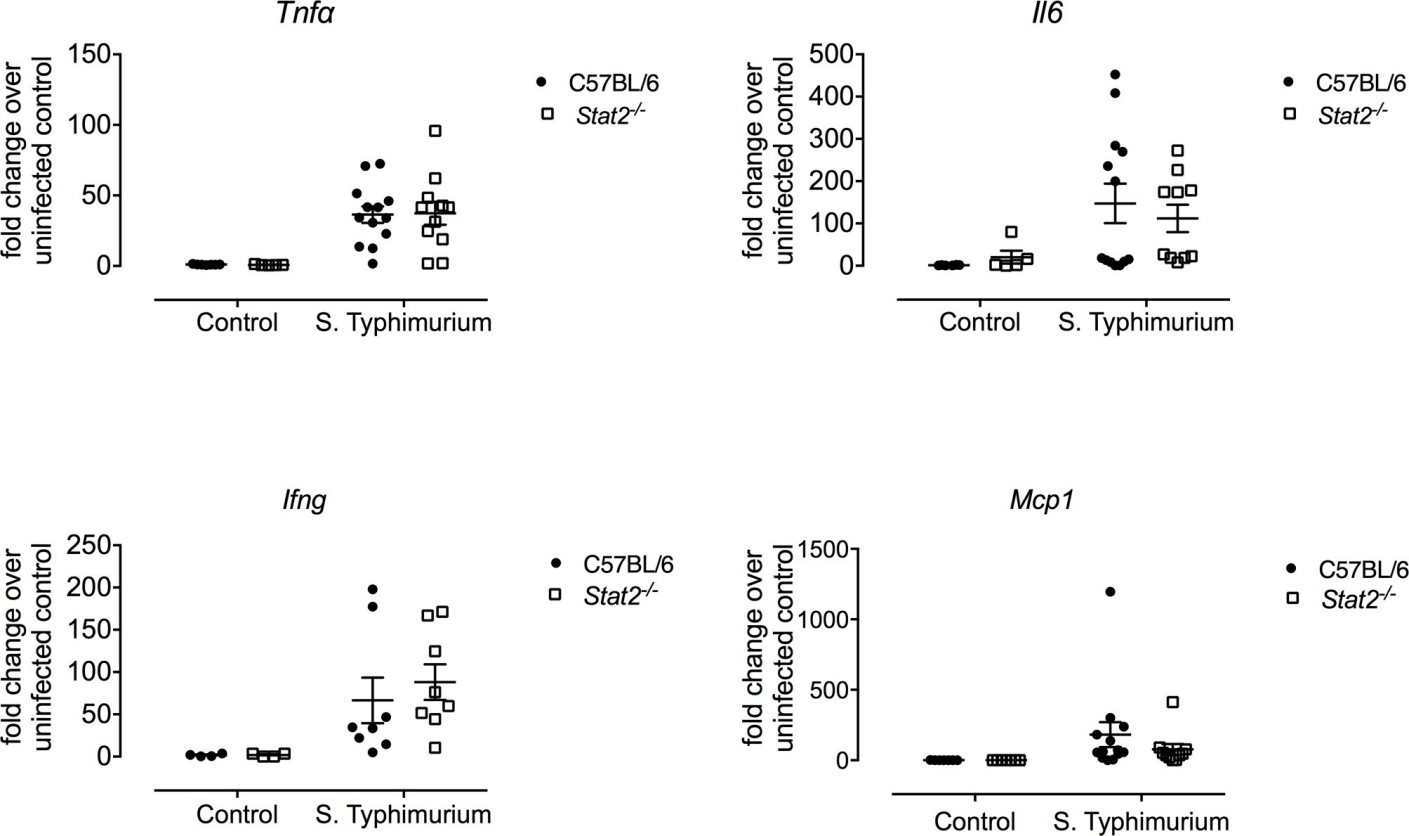
Transcript levels of genes classically associated with *S.* Typhimurium infection in ceca of C57BL/6 and *Stat2^-/-^* mice 48 hours after *S.* Typhimurium infection. C57BL/6 and *Stat2^-/-^* mice were orally infected with 10^9^ STM following streptomycin pretreatment. Expression levels of *Tnfα, Il6 Ifnγ* and *Mcp^1^* were determined by qPCR in the cecum of infected mice 48 post infection. Data were normalized to uninfected mice from each group. Each data point represents one analyzed mouse sample. Mean and SE were calculated by averaging results from three independent experiments. *p <0.05 as determined by Students t-test.

To determine the role of STAT2 during *S*. Typhimurium infection, we first evaluated intestinal immune responses by analyzing gene expression by qPCR in the cecum of mice at 48 hours post infection, a time point where no animal death was observed and found to be optimal for investigating inflammatory responses [36]. When we examined the expression of genes that have previously been identified to be dependent on STAT2 [37], we found that there were significantly lower transcript levels of *Irf7*, *Isg15*, *Oas1b*, *Rsad1*, and *IrgM1* in the cecum of infected *Stat2*^*-/-*^ mice when compared to cecum of infected wild-type mice (**Fig 3**). We found no significant differences in genes known to be regulated by IFN γ and the IFNGR such as *Cxcl10* (**Fig 3**). Furthermore, no differences in the transcription levels of genes previously shown to be important for *S*. Typhimurium infections including *Tnfα*, *Il6*, *Ifnγ*, and *Mcp1* were observed between wild-type and *Stat2*^*-/-*^ mice (**Fig 4**). This result was further confirmed when we analyzed the systemic cytokine responses in the serum using a cytometric bead assay. We did not observe a significant difference in the serum levels of TNFα, IFNγ, MCP1 (also known as CCL2), IL-12, IL-6 or IL-10 between wild-type and *Stat2*^-/-^ infected mice (S1 **Fig**). Overall, these results show that type I IFN signaling is distinctively blocked in *Stat2*^*-/-*^ mice as classical inflammatory gene expression was unaffected by this deficiency.

### Neutrophil-associated oxygenation contributes to low bacterial burdens in the guts of *Stat2*^*-/-*^ mice

**Fig 5:**
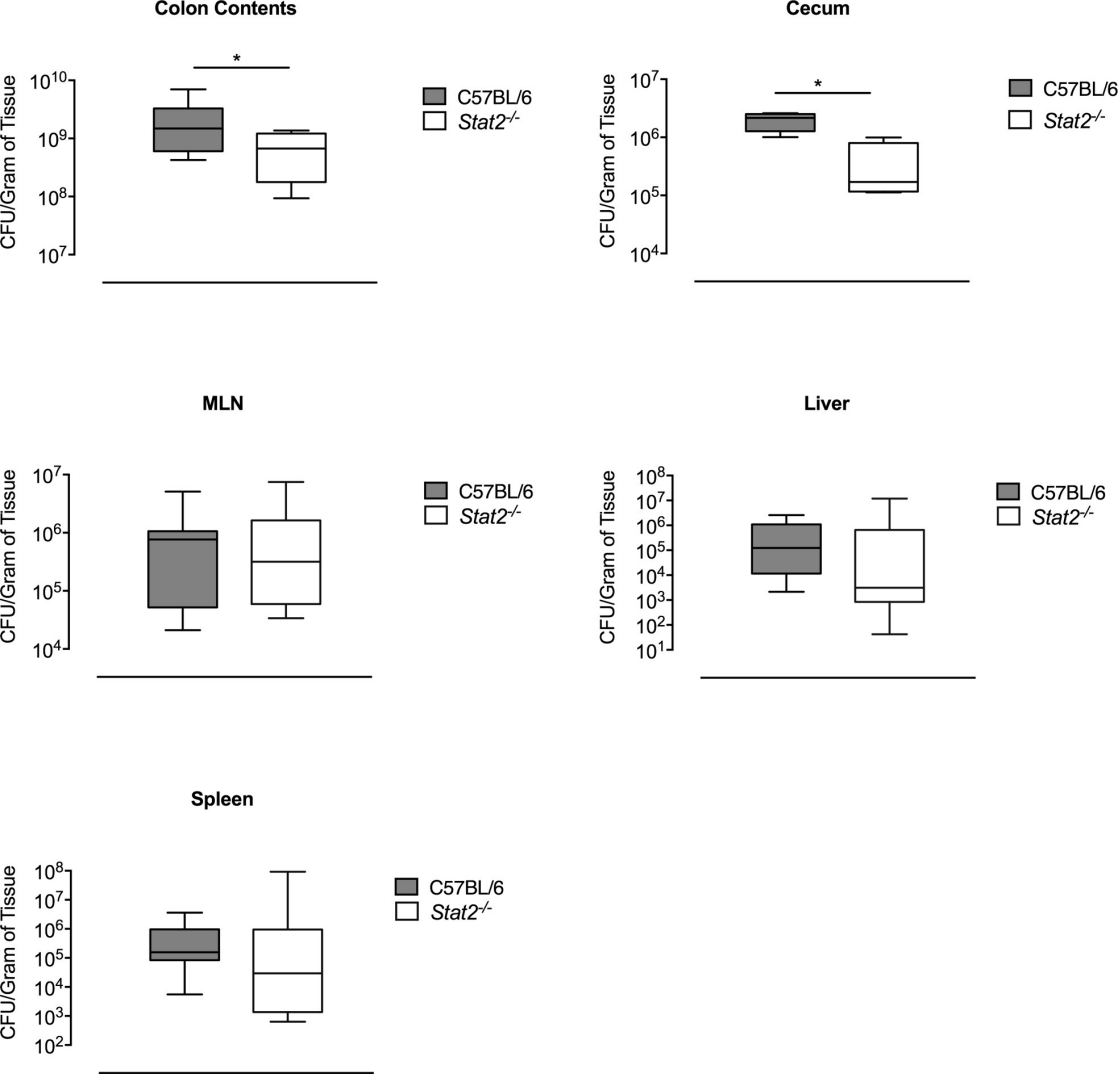
Bacterial burdens in various organs in C57BL/6 and *Stat2^-/-^* infected mice following *S.* Typhimurium infection. C57BL/6 and *Stat2^-/-^* mice were orally infected with 10^9^ CFU of *S.* Typhimurium following streptomycin pretreatment. Forty-eight hours following infection mice, were euthanized and the colon contents, cecum, mesenteric lymph nodes (MLN), and spleen were collected and plated for bacterial enumeration. Mean and SE were calculated by averaging results from three independent experiments. *p <0.05 as determined by Students t-test.

When we investigated the bacterial burdens at 48 hours post infection, the time point where we observed changes in immune responses, we found that there were significantly fewer bacteria in the cecum and colon contents of *Stat2*^*-/-*^ mice compared to wild-type mice (**Fig 5**). No differences were observed in bacterial numbers in mesenteric lymph nodes (MLN). Although there was a trend towards lower numbers in spleen and liver at this time point (**Fig 5**), it was not statistically significant. The fact that a deficiency in STAT2 signaling leads to decreased bacterial burden specifically in the lumen suggests a role for a STAT2-induced inflammatory environment in *S*. Typhimurium expansion.

**Fig 6:**
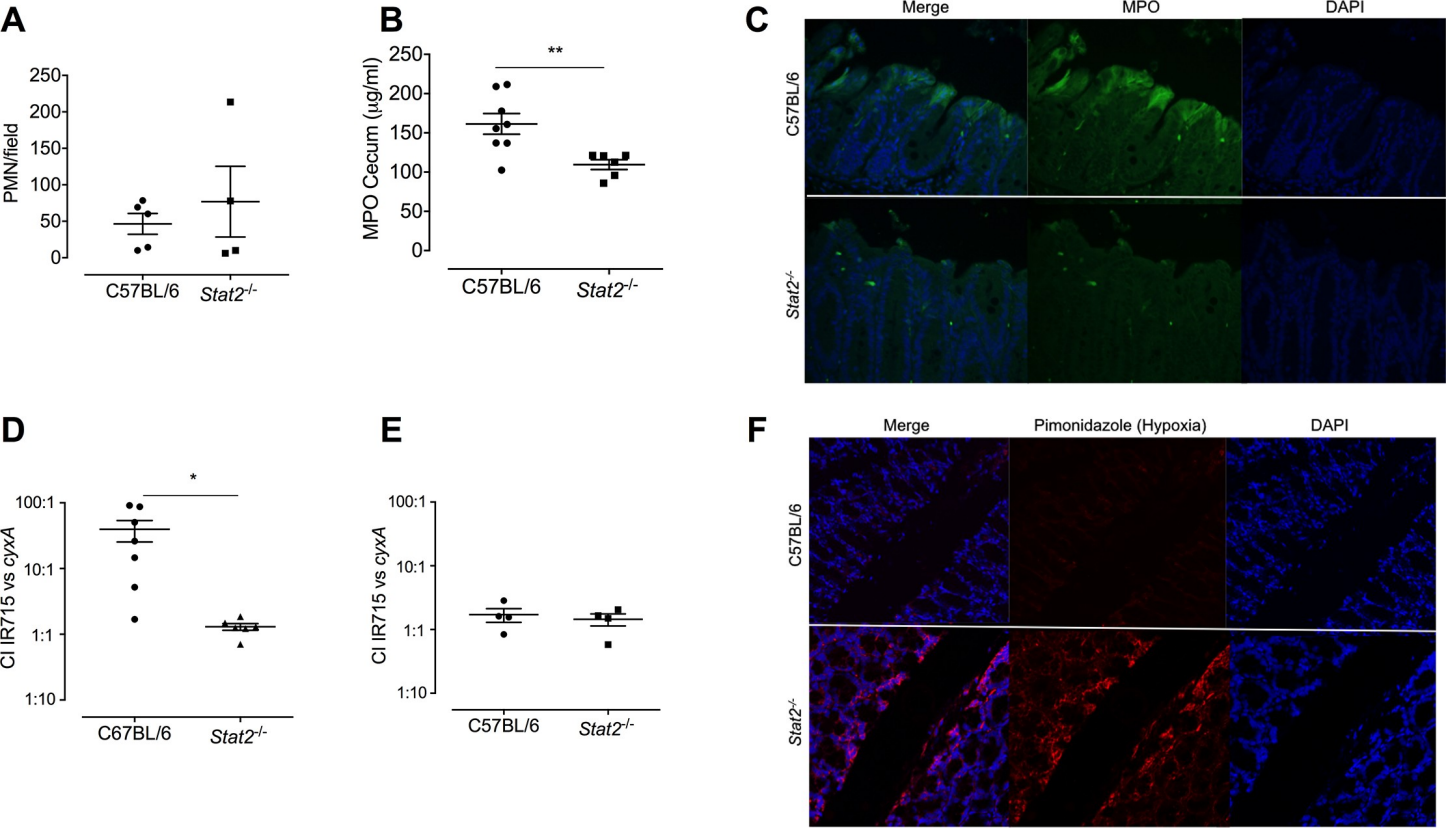
Neutrophil influx and luminal oxygenation promotes *S.* Typhimurium survival. C57BL/6 and *Stat2^-/-^* mice were orally infected with 10^9^ CFU of *S.* Typhimurium following streptomycin pretreatment. A. Neutrophils were counted in ten fields in H&E stained tissue sections and the number of neutrophils were averaged for each sample. B. The enzymatic activity of MPO was quantified in fecal samples by ELISA. C. Paraffin embedded sections were stained with anti-MPO antibody (green) as well as DAPI (blue) to mark the nucleus. Images were captured using SPOT imaging software at 40x. C57BL/6 and *Stat2^-/-^* mice were orally infected with a 1:1 ratio of wild type *S.* Typhimurium and *cyxA* mutant mutant following streptomycin pretreatment. Mice were euthanized 4 days after infection and the competitive index (CI, output ratio of WT/*cyxA* mutant divided by input ratio of WT/*cyxA* mutant) was calculated in the D. colon contents and E. liver. F. colons of WT and *Stat2^-/-^* mice infected with 1:1 ratio of WT *S.* Typhimurium: *cyxA* mutant were collected 4 days post infection and paraffin embedded. Tissues were stained for hypoxia using the pimonidazole hypoxia probe (red) and DAPI (blue). Images were captured at 63x using Leica confocal microscope. Mean and SE were calculated by averaging results from three independent experiments. *p <0.05, ** p<0.01 as determined by Students t-test.

In response to infection with *S*. Typhimurium, neutrophils migrate into the tissue as well as the lumen [38, 39]. Studies using different pathogens have suggested that type I IFNs not only mediate the migration of neutrophils into the infection site but also enhance their function [40, 41]. To determine whether there was a defect in neutrophil migration as well as pathology, cecal tissue samples were fixed and stained with H&E. No differences were observed in overall histopathology between wild-type and *Stat2*^*-/-*^ mice infected with wild-type *S*. Typhimurium at 48 hours (**S2 Fig**). Neutrophil numbers (PMN/field) were similar between wild-type and *Stat2*^*-/-*^ mice infected with wild-type *S*. Typhimurium (**Fig 6A).** No differences in neutrophil abundance were noted when comparing uninfected wild-type and *Stat2*^*-/-*^ (**S3A Fig).** However, when we quantified levels of myeloperoxidase (MPO), a neutrophil marker, we surprisingly found that there was less MPO in the cecal tissue of *S*. Typhimurium-infected *Stat2*^*-/-*^ mice than infected wild-type mice (**Fig 6B**). This result was confirmed using immunohistochemistry with an MPO-specific antibody (**Fig 6C**). These results suggested that although the presence of type I IFNs does not affect the transmigration of neutrophils into the infection site, they somehow alter the function of these immune cells. This finding is somewhat surprising because neutrophils play a major role in clearing *S*. Typhimurium. The fact that there were more bacteria in the presence of neutrophils indicated to us that a novel mechanism allows this pathogen to thrive in the presence of neutrophils.

To determine if intestinal oxygenation caused the difference in bacterial burdens in the intestines of wild-type versus the *Stat2*^*-/-*^ mice, we infected mice with the *S*. Typhimurium *cyxA mutant*. The *cyxAB* operon encodes a cytochrome *bd-*II oxidase enzyme that facilitates growth of *S*. Typhimurium under oxygen-limiting conditions [42–45]. CyxA is essential for *S*. Typhimurium survival in the post-antibiotic treatment model [45]. Mice were streptomycin pretreated and then orally administered a 1:1 mixture of wild-type *S*. Typhimurium and *cyxA* mutant. Four days post infection colon contents were collected for bacterial enumeration by determining colony forming units (CFU), and the competitive index (CI) was determined by dividing the output ratio (wild-type CFU/*cyxA* CFU) in the colonic contents of mice by the input ratio (wild-type CFU/*cyxA* CFU). Wild-type *S*. Typhimurium exhibited a fitness advantage over the *cyxA* mutant in wild-type mice (higher numbers of wild-type bacteria recovered), consistent with previous findings [45]; however, the *cyxA* gene provided no advantage in the *Stat2*^*-/-*^ mice (both strains were recovered at same numbers) (**Fig 6D**). There was no observable phenotype in systemic sites such as the liver where there was no fitness advantage conferred by the *cyxA* mutant in either the wild-type or STAT2^-/-^ mice (**Fig 6E**). These data suggested that oxygenation in the intestine of wild-type mice is different from that of *Stat2*^*-/-*^ mice. This was confirmed using pimonidazole (PMDZ), a marker of hypoxia. Mice were injected intraperitoneally with PMDZ (Chemicon; 2.0 mg/20 g body weight in 100 μl PBS) 1 hour prior to euthanasia, and PMDZ was detected in tissue sections by immunohistochemistry. It was previously reported that hypoxia decreases in the intestine during *S*. Typhimurium infection [45]. We determined that the intestinal environment in *Stat2*^*-/-*^ mice was more hypoxic than in wild-type mice as shown by higher levels of pimonidazole staining (red) (**Fig 6F).** Both wild-type and *Stat2*^*-/-*^ mice showed comparable hypoxia staining without infection (**S3B Fig**).

### Specific depletion of Bacteroidetes during *S*. Typhimurium infection is STAT2 dependent

**Fig 7:**
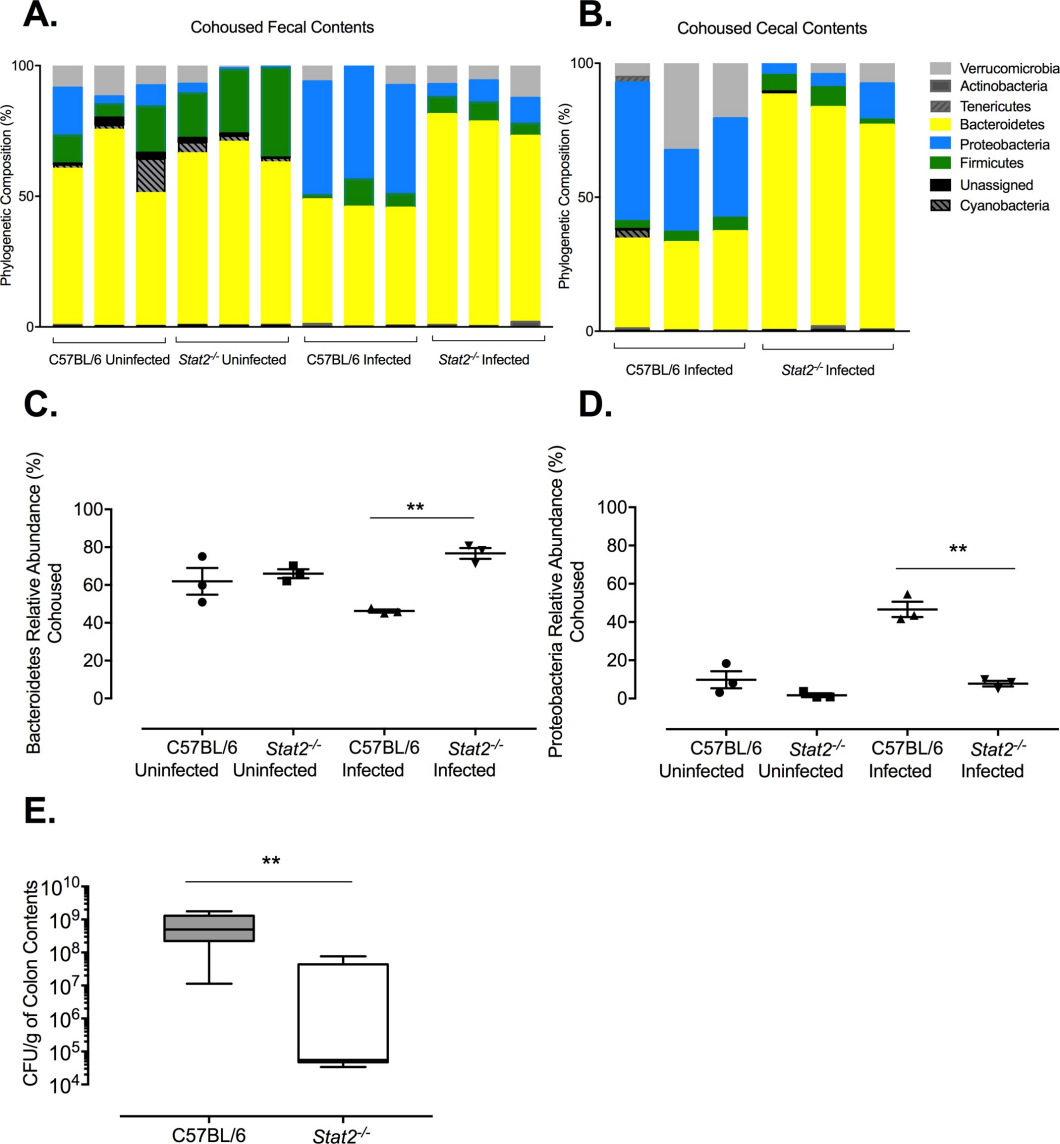
16S rRNA profiling of the microbiome from the *S.* Typhimurium infected wild-type and *Stat2^-/-^* mice following 5 weeks of co-housing. A. Phylum level microbiota composition was determined in uninfected and infected C57BL/6 and *Stat2^-/-^* mice through 16S rRNA analysis in fecal DNA samples as well as B. cecal DNA samples. C. Relative abundance of Bacteroidetes was determined in fecal DNA samples from uninfected and infected C57BL/6 and *Stat2^-/-^* mice D. Relative abundance of Proteobacteria was determined in fecal DNA samples from uninfected and infected C57BL/6 and *Stat2^-/-^* mice. E. CFU/g of *S.* Typhimurium was enumerated in the colon contents of C57BL/6 and *Stat2^-/-^* mice 48 hours post infection. Mean and SE were calculated by averaging results *p <0.05, as determined by Students t-test.

Obligate anaerobes of the healthy gut microbiota were previously reported to become depleted from the microbiota at later stages of *S*. Typhimurium infection in streptomycin-treated mice through a neutrophil-dependent mechanism [46]. To determine if STAT2 signaling led to changes in the microbiota, we analyzed the phylogenetic composition of the intestinal microbial communities using 16S rRNA profiling (**S4A Fig**). We observed a drastic reduction in the relative abundance of Bacteroidetes phylum with approximately 10% remaining in wild-type mice infected with *S*. Typhimurium as opposed to 60% in uninfected wild type mice. No significant shifts were detected in the abundance of Bacteroidetes in *Stat*2^-/-^ infected mice when compared to that of uninfected wild-type and *Stat2*^-/-^ control mice (**S4B Fig**). Conversely to the CFU recovered from feces of wild-type and *Stat2*^-/-^ mice upon *S*. Typhimurium infection (**Fig 5**), there was a significantly higher relative abundance of Proteobacteria observed in the wild-type infected mice with an average of 70% than in the *Stat2*^*-/-*^ infected mice (30%; **S4C Fig**). To verify that the experimental changes we observed between wild-type and *Stat2*^-/-^ mice was not due to differences in the overall microbiome content of the two strains of mice that arose because they were housed separately, we co-housed the mice starting at the day of weaning for 5 weeks. The co-housed wild-type and *Stat2*^-/-^ mice were infected with *S*. Typhimurium following streptomycin pre-treatment. Forty-eight hours post infection, fecal and cecal contents were collected and the phylogenetic composition of the microbial communities at the phylum level was determined using 16S rRNA profiling (**Fig 7A and 7B)**. Uninfected mice were also included as controls. Infection of wild-type mice with *S*. Typhimurium led to a reduction in the relative abundance of the Bacteroidetes phylum. The relative abundance of Bacteroidetes in *Stat2*^*-/-*^ infected mice remained comparable to that of uninfected control mice (**Fig 7C).** It’s important to emphasize that the striking reduction in Bacteroidetes abundance we detected earlier in non-cohoused infected and non-infected wild type mice were no longer observed under co-housing conditions. Nevertheless, differences between infected wild type and *Stat2*^*-/-*^ mice remained unchanged. Moreover, relative abundance of Proteobacteria was increased in the wild-type infected mice and this expansion was at a lower level in *Stat2*^*-/-*^ infected mice (**Fig 7D)**. Detailed microbial analysis revealed that the members of Proteobacteria that expanded in their relative abundance in infected wild-type mice belonged to the *Salmonella* genus (**S5 Fig**). This result was validated by directly enumerating *S*. Typhimurium in the colon contents of co-housed infected wild-type and *Stat2*^*-/-*^ mice (**Fig 7E**). Overall, the results obtained in both experiments using co-housed and non-cohoused mice demonstrated that STAT2 enabled the expansion of *S*. Typhimurium. These data also strongly indicate that post-infection, *Stat2*^*-/-*^ mice retained a protective microbiome against pathogenic bacteria.

**Fig 8:**
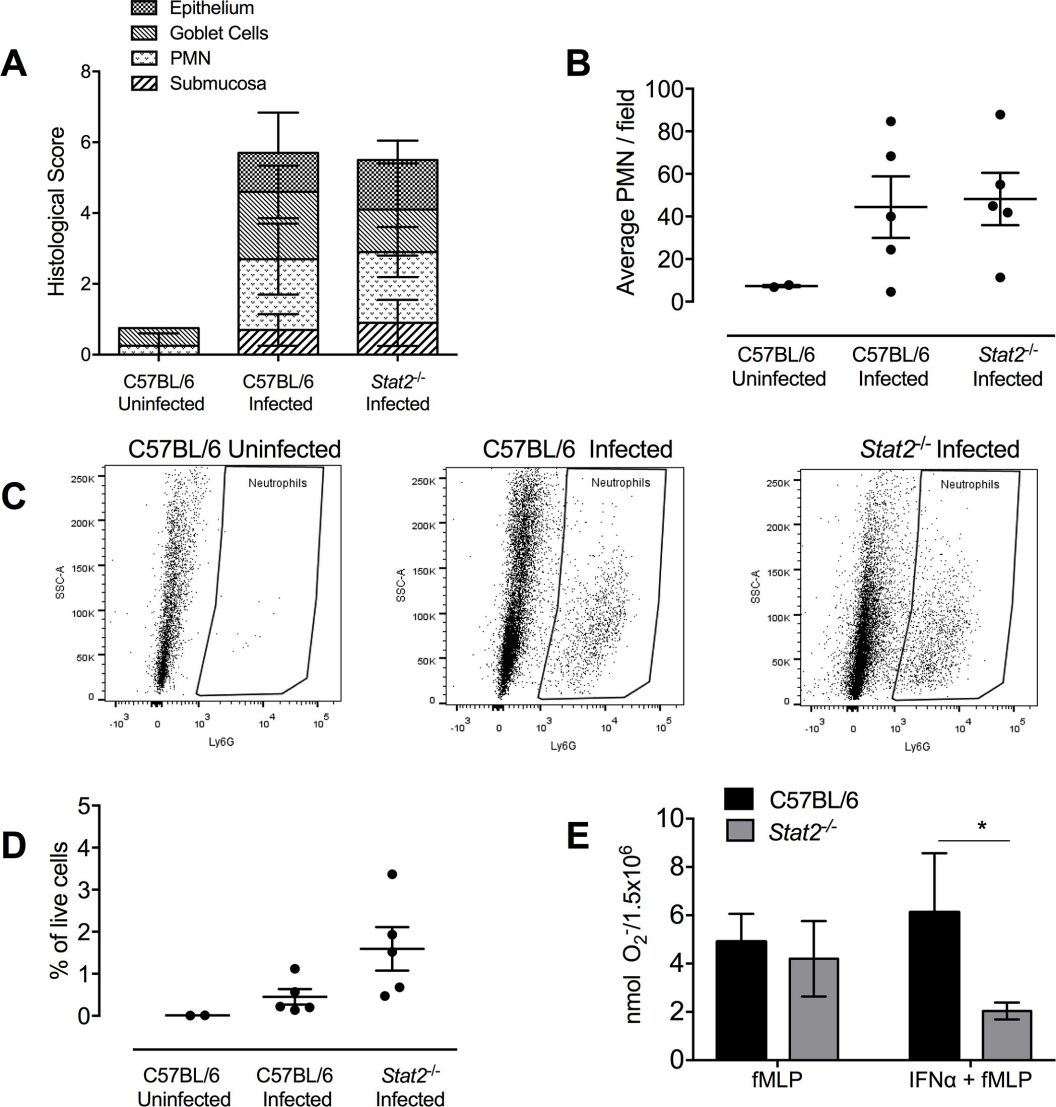
Neutrophil analysis in co-housed wild-type and *Stat2^-/-^* mice infected with *S.* Typhimurium. A. for the cecum of uninfected and *S.* Typhimurium infected wild-type C57BL/6 and *Stat2^-/-^* mice. B. Average neutrophils (Cumulative histopathology score PMN) numbers enumerated in ten fields from cecum of uninfected and *S.* Typhimurium infected wild-type C57BL/6 and *Stat2^-/-^* mice. C. Flow cytometry analysis of cecal cell suspensions. Neutrophils were gated after duplet and dead cell elimination for the following markers; CD45^+^, CD3^-^, CD56^-^, CD19^-^, Ly6G^+^ D. Percent of live cells as determined by flow cytometry for C57BL/6 uninfected, C57BL/6 *S.* Typhimurium infected and *Stat2^-/-^* *S.* Typhimurium infected mice. E. Superoxide anion (O_2_^-^) generation in response to fMLP in the presence or absence of IFNα by bone marrow neutrophils isolated from wild-type and *Stat2^-/-^* mice. Mean and SE were calculated by averaging results from three independent experiments. *p <0.05, ** p<0.01 as determined by Students t-test.

To also confirm the previous findings on neutrophils (**Fig 6**), we performed a semi-quantitative analysis of overall pathology and quantified neutrophil abundance. There were no differences in the overall pathology (**Fig 8A**) and the neutrophil numbers between the *S*. Typhimurium infected wild-type and *Stat2*^*-/-*^ mice (**Fig 8B**). The cecal neutrophils were also quantified using flow cytometry. Following the identification of live cells, neutrophils were identified as CD45^+^, CD3^-^, NK1.1^-^, B220^-^, Ly6G^+^ cells using the gating strategy described in **S6 Fig**. While there was an increase in percentage of neutrophils in wild-type mice infected *S*. Typhimurium compared to uninfected wild-type mice, there were no significant difference observed in the percentage of neutrophils when comparing *S*. Typhimurium infected wild-type and with *S*. Typhimurium infected *Stat2*^*-/-*^ mice (**Fig 8C**). As we did not observe any differences between numbers of neutrophils transmigrating into the infection site between wild-type and *Stat2*^*-/-*^ mice but there was a difference in MPO levels in the colon contents of these mice (**Fig 6C**), we next determined whether *Stat2*^*-/-*^ neutrophils were functional. Bone marrow neutrophils from wild-type and *Stat2*^*-/-*^ mice were stimulated with the Gram-negative bacterial ligand *N*-formylmethionyl-leucyl-phenylalanine (fMLP) in the absence or presence of IFNα. Superoxide anion generation was then measured. There were no differences in superoxide anion generation between the neutrophils of wild-type and *Stat2*^*-/-*^ mice upon stimulation with fMLP. However, when the cells were pre-treated with a type I IFN, IFNα, there were reduced levels of superoxide anion generation in the neutrophils isolated from *Stat2*^*-/-*^ mice compared to wild-type mice (**Fig 8E**)

## Discussion

The immune system deploys multiple mechanisms to eradicate invading microbes and infections. Induction of type I IFNs is a critical mechanism that the immune system exploits to fight viral infections. Type I IFNs (IFNs α and β) induce antiviral responses by binding to their cognate receptor IFNAR ubiquitously expressed on many cell types. The transcription factor STAT2 takes center stage in the type I IFN response as it is essential to mediate an antiviral state that helps the host clear a viral infection [47]. Research over the past few years has suggested that type I IFNs are intricate players during bacterial infections. Although type I IFN responses mounted against viral infections provide a common anti-viral state among a broad range of viral pathogens, the type I IFN response generated against bacteria varies based on the specific bacterial pathogen. Recently, it was reported that *Ifnβ*^*−/−*^ mice exhibit greater resistance to oral *S*. Typhimurium infection and a slower spread of *S*. Typhimurium to distal sterile sites [30]. These results are consistent with our findings using *Stat*2^-/-^ mice (**Fig 2**). Nevertheless, the previous study did not use streptomycin pre-treatment to induce colitis during infection, which models more accurately the course of *S*. Typhimurium infection. Hence the role of type I IFNs during gut inflammation and dysbiosis has remained unclear. Several studies have emerged showing that not all type I IFN responses involve the classical ISGF3 complex. STAT2 homodimers have been shown to bind IRF9 and activate ISG expression of antiviral genes in the absence of STAT1 [37, 48]. The expression of a subset of ISGs stimulated by STAT2 homodimers/IRF9 exhibits a delayed kinetics compared to the classical ISGF3, however, this is sufficiently robust to evoke an innate response [49]. These observations together with our own findings indicate that *S*. Typhimurium exploits the type I IFN pathway by relying on STAT2 and potentially in the absence of STAT1.

*S*. Typhimurium successfully establishes an infection with the coordinated actions of its two distinct populations; the first invades the tissue and increases inflammation while the second luminal population counter intuitively benefits from the generation of host derived nitrate and oxygen [14, 15, 50]. The regulatory host signaling pathways that control the availability of these electron acceptors are not known. Here, we determined that type I IFN pathway is activated during *S*. Typhimurium infection and leads to oxygenation of the gut mucosa allowing the pathogen to respire and expand its luminal population. As we observed blunted expression of type I IFN stimulated genes (Fig 3), similar numbers of neutrophils in the cecal mucosa but lower levels of MPO (Fig 6A and 6B), a neutrophil activation marker, in the cecum of *Stat2*^*-/-*^ mice infected with *S*. Typhimurium, these results suggest that type I IFNs do not effect the migration of neutrophils to the site of infection but may effect the antimicrobial activity of these cells. It was previously established that upon activation, neutrophils release reactive oxygen species as antimicrobial measures. The release of reactive oxygen species also contributes to oxygenation of the lumen, and superoxide dismutases encoded by *Salmonella* allow the bacteria to detoxify the oxygen radicals promoting bacterial survival [51]. The competition experiments between wild-type *S*. Typhimurium and *cyxA* mutant as well as hypoxia staining confirmed that oxygenation of the gut lumen of *Stat2*^*-/-*^ mice was lower compared to that of wild-type mice. Furthermore, our *in vitro* experiments confirmed that neutrophils from the *Stat2*^*-/-*^ mice were blunted in their ability to generate superoxide anion. Overall, these results suggest that in response to *S*. Typhimurium, neutrophils invade the gut lumen and contribute to the oxygenation of the gut via a type I IFN mediated mechanism. In return, the professional pathogen *S*. Typhimurium takes advantage of this mechanism and expands its population.

One of the many benefits of the gut microbiota to the host is to limit the expansion of enteric pathogens. Gut microbiota provides metabolites such as butyrate that fuels colonocyte metabolism resulting in the consumption of oxygen, thereby rendering the lumen hypoxic. It was recently shown that the epithelial PPAR-γ-signaling pathway limits oxygenation of the gut epithelium in the presence of butyrate, which in turn limits the expansion of *S*. Typhimurium [50]. In addition to the neutrophils, we do not know whether STAT2 signaling may also have a direct effect on colonocyte metabolism (**Fig 6D**), which impacts the bioavailability of oxygen in the gut lumen. Previous studies have shown that the abundance of dominant microbial phyla, Bacteroidetes and Clostridia can directly be affected by drastic changes in the luminal environment during enteric infections [52–54]. The depletion of Clostridia from the microbiota at later stages of *S*. Typhimurium infection in streptomycin-treated mice through a neutrophil-dependent mechanism was reported [46]. Our results demonstrate that a STAT2 mediated type I IFN response triggered during *S*. Typhimurium infection directly affects Bacteroidetes phyla in the gut.

Our study highlights the importance of STAT2 signaling in neutrophils during *Salmonella* infection. To date, most of what has been described for STAT2 signaling in pathogenic infections was centered on immune cells, such as macrophages and dendritic cells. In models of viral infection, STAT2 signaling is exploited by measles virus and choriomeningitis virus to interfere with dendritic cell (DC) development and expansion [55]. Furthermore, STAT2 signaling in macrophages is critical to activate a transcriptional response and control early dengue virus replication [37, 56]. We speculate that STAT2 signaling in colonocytes during Salmonella infection is equally important as in neutrophils for crosstalk and the release of chemokines for the recruitment and activation of neutrophils and macrophages. Future studies involving a more detailed analysis are warranted to delineate the far-reaching effects of type I IFN signaling on both the microbiota and oxygenation by colonocytes and neutrophils.

## Materials and methods

### Bacterial strains

*Salmonella enterica* serovar Typhimurium strain IR715, a fully virulent, spontaneous nalidixic acid resistant derivative of strain ATCC 14028, was grown in Luria-Bertani broth (LB) supplemented with 50 μg/ml nalidixic acid at 37°C [57]. *Salmonella* Typhimurium IR715 *cyxA* [45], generously provided by Andreas Baumler, was supplemented with 100 μg/ml carbenicillin LB broth and was grown at 37°C.

### Experimental infection of mice

Eight- to ten-week-old female C57BL/6 (wild-type) mice were age and sex matched to mice deficient in STAT1 (kindly provided by Dr. David Levy, NYU) or STAT2 (generously provided by Dr. Christian Schindler on the SvJ background that we backcrossed 10 generations onto the B6 genetic background). All mice were bred at the animal facility of the Lewis Katz School of Medicine at Temple University. All mice were streptomycin treated prior to bacterial infection. Mice were monitored twice daily after infection. Humane terminal endpoints included inability to ambulate and/or labored breathing. Briefly, mice were inoculated intragastrically with 20 mg of streptomycin (0.1 ml of a 200 mg/ml solution in water) 24 hours prior to bacterial infection. Bacteria were grown with shaking in LB broth containing nalidixic acid (50 μg/ml) at 37°C overnight. For infection, groups of 3 to 5 mice were inoculated intragastrically with either 0.1 ml of sterile LB broth (mock infection) or 10^9^ CFU of *S*. Typhimurium. Mice were sacrificed at indicated time points after infection. To determine the number of viable *S*. Typhimurium, samples of cecum (proximal section), liver, spleen, mesenteric lymph nodes, and colon contents were collected from each mouse and homogenized in 5 ml PBS. 10-fold serial dilutions were plated on LB agar plates containing nalidixic acid (50 μg/ml). The tip of the cecum was collected for histopathological analysis. The center section of the cecum was immediately snap-frozen in liquid nitrogen and stored at −80°C for RNA isolation. All animal experiments were repeated at least three times with identical results.

To determine to role of luminal oxygenation in bacterial survival, 24 hours prior to inoculation 6 to 8 week-old age matched wild-type C57BL/6 and *Stat2*^*-/-*^ mice were orally gavaged 0.1 ml of a 200mg/ml streptomycin solution. Mice were orally infected with 10^8^ bacteria in a 1:1 ratio of *S*. Typhimurium IR715:*cyxA*. Four days after infection mice were euthanized and colon contents, cecum and liver were collected to determine the CFU of IR715 and *cyxA* mutant. The cecum was snap frozen in liquid nitrogen and stored at -80°C for MPO ELISA, and sections of the colon were collected for histopathological analysis. Organs for bacterial enumeration were homogenized as mentioned above and plated on selective media using 10-fold serial dilutions. The competitive index (CI) was calculated as the ratio of recovered bacterial strains (output ratio) divided by the ratio present in the inoculum (input ratio). All animal experiments were at least repeated three times with identical results.

### Quantitative real time PCR

RNA was extracted from snap-frozen tissues or tissue culture cells using 1 ml TriReagent (Molecular Research Center, TR118) according to the manufacturer's protocol. RNA was then treated with DNase according to the manufacturer’s protocol (Ambion, AM1906). Reverse transcription of total RNA (1 μg) was performed in 25 μl volume according to manufacturer's instructions using the TaqMan Reverse Transcription Kit (Invitrogen, N8080234). Real-time PCR was performed using the SYBR green (Applied Biosystems, 4309155) or TaqMan (Applied Biosystems) according to the manufacturer's instructions. Real-time PCR was performed for each cDNA sample (5 μl per reaction) in duplicate using the Step One Plus real-time PCR system (Applied Biosystems). The primers sequences are listed in Table 1. Results were analyzed using the comparative ΔC_T_ method. Data was normalized to *Gapdh* or β*-actin* for SybrGreen or TaqMan reagents, respectively. Fold increases in gene expression in infected or mock-infected *Stat2*^*-/-*^ mice were calculated relative to the average level of the respective cytokine in the mock-infected wild-type mice.

**Table 1:** Primers used for qPCR.

Gene Target	Direction	Sequence	Source
*Irf7*	Forward	5' CAG CAG TCT CGG CTT CTG 3’	35
	Reverse	5' TGA CCC AGG TCC ATG AAG TG 3’	
*Irgm1*	Forward	5’ TGCTCCACTACTCCCCAACAT 3’	Harvard Primer Bank
	Reverse	5’ GCTCCTACTGACCTCAGGTAAC 3’	
*Isg15*	Forward	5' GGT CTC CGT TAA CTC CAT 3’	Harvard Primer Bank
	Reverse	5' TCC AAA GGG TAA CAC CGT CCT 3’	
*Oas1b*	Forward	5’ GGGCCTCTAAAGGGGTCAAG 3’	Harvard Primer Bank
	Reverse	5’ TCAAACTTCACTCCACAACGTC 3’	
*Rsad1*	Forward	5’ GTACCACTGTGACGACCACC 3’	Harvard Primer Bank
	Reverse	5’ TATTGGCGAAAGCCAGCATCT 3’	
*Cxcl10*	Forward	5’ CCA AGT GCT GCC GTC ATT TTC 3’	Harvard Primer Bank
	Reverse	5’ GGC TCG CAG GGA TGA TTT CAA 3’	
*Ifnγ*	Forward	5’ TCTCCAGAAACCCTCACTGGT 3’	Harvard Primer Bank
	Reverse	5’ TCAGCGGATTCATCTGCTTCG 3’	
*Mcp1*	Forward	5’ AAAACACGGGACGAGAAACCC 3’	Harvard Primer Bank
	Reverse	5’ ACGGGAACCTTTATTAACCCCT 3’	
*Tnfα*	Forward	5’ CCC TCA CAC TCA GAT CAT CTT CT 3’	Harvard Primer Bank
	Reverse	5’ GCT ACG ACG TGG GCT ACA G 3’	
*Il-6*	Forward	5’ GGTGCCCTGCCAGTATTCTC 3’	Harvard Primer Bank:
	Reverse	5’ GGCTCCCAACACAGGATGA 3’	
*Gapdh*	Forward	5’ CCA GGA AAT CAG CTT CAC AAA CT 3’	36
	Reverse	5’ CCC ACT CCT CCA CCT TTG AC 3’	
*β-actin*	Forward	5’GAGTCCTACGACATCATCGCT 3’	Harvard Primer Bank
	Reverse	5’ CCGACATAGTTTGGGAAACAGT 3’	

### Hypoxia staining

Hypoxia studies were performed as described by the manufacturer’s instructions (Hypoxyprobe-1 Plus Kit, Chemicon, Temecula, CA, USA) [45]. One hour prior to euthanasia, wild-type and *Stat2*^*-/-*^ infected mice were injected with 100 mg/kg of PMDZ diluted in DMSO. After euthanasia, colon samples were collected and fixed with 10% formalin. Unstained paraffin embedded tissue samples were probed with 1:50 FITC-conjugated IgG_1_ mouse monoclonal antibody clone 4.3.11.3 (Hypoxyprobe, Inc.), and stained with 1:150 Cy3 conjugated AffniPure Goat Anti Mouse IgG (H+L) (Jackson ImmunoResearch, 115-165-0003). Briefly, tissue sections were incubated at 50°C for 10 minutes and then deparaffinized by washing for 10 minutes with xylene 2x, 3 minutes with 95% ethanol 2x, 3 minutes with 80% ethanol 1x, and then rehydrated by washing with 70% ethanol 1x. The antigens were retrieved by incubating sections with 20μg/ml Proteinase K (Fisher, BP1700-100) in TE buffer (10mM Tris, 1mM EDTA, pH 8.0) for 15 min at 37°C in a humidified chamber. The slides were washed with PBS for 10 minutes and then blocked for 45 minutes with blocking buffer. Samples were incubated with the primary antibody 1: 50 FITC-conjugated IgG_1_ mouse monoclonal antibody clone 4.3.11.3 over night at 4°C in a humidified chamber. After PBS washing (5 minutes, trice), each slide was incubated with the secondary antibody 1:150 Cy3 conjugated AffniPure Goat Anti Mouse IgG (H+L) at room temperature for 90 minutes in a humidified chamber. DAPI (Invitrogen, P21490) was used as a counter-stain (1μg/ml, incubated at room temperature for 5 minutes in the dark). Slides mounted with Vectashield (Vector Labs, H-1000) and were visualized using an Olympus BX60 Fluorescent Microscope with Spot Insight2 camera at 10x magnification.

### Myeloperoxidase (MPO) activity assay

MPO activity in the cecal tissue was determined as previously described [58]. Snap frozen cecum samples were lysed by homogenizing in 0.5% HETAB (hexadecyltrimethyl ammonium bromide) in 50mM KPi (phosphate buffer) at a ratio of 0.1g sample per 1ml buffer. Master mixes were prepared by mixing 10 μL homogenized sample with 3 μL o-dianisidine hydrochloride (20mg/ml stock), 3 μL 20 mM hydrogen peroxide and 284μL 50mM KPi. Ten-fold serial dilutions of the MPO standard 100UG (Millipore, 475911) were prepared in the same fashion as the samples, with the top standard being 125μg/ml. Samples were plated in clear 96 well plate and incubated at 37°C for 10 minutes, taking absorbance measurements at 460 nm every 2 minutes for 10 minutes using a Flex Station, Molecular Devices plate reader. The reaction was halted by adding 3 μL of 30% NaN_3_ to each well and a final absorbance reading at 460nm was taken. The concentration of MPO was calculated using the absorbance values obtained from the standard curve.

### Myeloperoxidase (MPO) staining

To visualize the presence of MPO within the cecum of wild-type C57BL/6 and *Stat2*^*-/-*^ mice, unstained paraffin embedded tissue sections were heated at 55–60°C for 30 minutes. The tissue was deparaffinized by washing in xylene 2x for 5 minutes, absolute ethanol 3x for 3 minutes, 95% ethanol 1x for 3 minutes, 90% ethanol 1x for 3 minutes, 70% ethanol 1x for 3 minutes. Antigens were retrieved by boiling slides in sodium citrate buffer (10mM Sodium Citrate, 0.05% Tween 20, pH 6.0), for 10 minutes, and allowing to cool to room temperature for 20 minutes. The samples were washed in Tris Buffered Saline, TBS (0.5M Tris Base, 9% NaCl) 1x for 5 minutes. The tissue was blocked in TBS supplemented with 3% BSA for 30 minutes and then incubated with 1:200 MPO Heavy Chain (L-20), (Santa Cruz sc-16129) in TBS supplemented with 3% BSA for two hours at room temperature. The samples were washed 3x for 5 minutes each in TBS supplemented with 3% BSA. The samples were then incubated for 40 minutes at room temperature with 1:1000 Rabbit anti-Goat (H+L) Super Clonal Secondary antibody, Alexa Fluor 488 conjugated (Fisher A27012). The slides were washed with 3x for 5 minutes each in TBS supplemented with 3% BSA. Samples were counter stained with 1 μg/ml DAPI (Invitrogen, P21490) and then washed in TBS supplemented with 3% BSA [59]. Slides were mounted with Vectashield (Vector Labs, H-1000) and visualized using Olympus BX60 Fluorescent Microscope with Spot Insight2 camera at 10x magnification.

### Microbiota analysis

DNA from fecal contents of wild-type and *Stat2*^-/-^ infected mice was extracted using the PowerSoil DNA Isolation Kit (MoBio, 12888–50) according to manufacturer’s protocol. High quality isolated DNA was then submitted to SeqMatic for 16S rRNA V4 sequencing using the Illumina MiSeq platform. Data was then analyzed using Qiime pipeline as described [2]. For the co-housing, wild-type (two or three) and *Stat2*^*-/-*^ mice (two or three) were placed in the same cages at the time of weaning and housed together for 5 weeks.

### Flow cytometry

The immune cells from the cecal tissue were isolated using the mouse *lamina propria* dissociation kit (Miltenyi Biotech, 130-097-410) according to manufacturer’s protocol. Briefly, 1x10^6^ cells were resuspended in PBS and stained with live/dead cell discriminator (Invitrogen, L34597) according to manufacturer’s protocol. Cells were then rinsed with PBS, spun down at 400g for 10 minutes and resuspended in 20ml of mouse Fc Block (Miltenyi,130-092-575). The cells were then incubated at room temperature for 15 minutes. The mouse Fc Block was left on the cells and the cells were then stained with CD45 PE-Cy7 Rat (Clone30-F11; Biolegend, 103114), CD3 APC Rat (Clone 17A2; Biolegend, 100236), B220 APC rat (Clone RA3-6B2, Biolegend, 103212), NK1.1 APC mouse (Clone PK136, Biolegend, 108710) and Ly6G Alexa 488 rat (Clone 1A8; Biolegend, 127626) diluted in fluorescence activated cell sorting (FACS) buffer according to the manufacturer’s instructions for 30 minutes at 4°C in the dark. The FACS buffer was composed of PBS, 0.5% BSA and 2% FBS. Cells were then rinsed with FACS buffer, spun down at 400g for 10 minutes and then resuspended in 100 ml of 4% paraformaldehyde (BD, 554655), which fixed the cells. Following a 20 min incubation in the dark at room temperature, the cells were washed with FACS buffer. Finally, the cells were spun down and resuspended in 400 ul of FACS buffer. Cells were analyzed on a BD FACS Canto flow cytometer (BD Biosciences) and analyzed using FlowJo software (TreeStar, Inc., Ashland, OR).

### Mouse bone marrow neutrophil isolation

Mouse bone marrow neutrophils were isolated according to the method of Mocsai et al. [60]. Wild-type and *Stat2*^*-/-*^ mice were euthanized and the femur and tibias from the hind legs harvested. Neutrophils were isolated by Percoll density gradient sedimentation, followed by hypotonic lysis to remove erythrocytes.

### Superoxide anion generation

Superoxide anion (O2^-^) generation was measured spectrophotometrically as superoxide-dismutase (SOD)-inhibitable cytochrome c reduction. In 96 well plates, bone marrow neutrophils (1.5 X 10^6^) from wild-type and *Stat2*^*-/-*^ mice were activated with fMLP (10^-8^M) in the presence of 5 μg/ml cytochalasin B. For experiments examining the effect of type I IFN on fMLP-stimulated O2^-^ generation, the neutrophils were pre-treated with IFNα (1000 units/ml) prior to the addition of fMLP. The generation of O2^-^ was monitored over a 10 min time-period [61, 62].

### Statistical analysis

For analysis of bacterial numbers, competitive indices, relative abundance of bacterial populations and fold changes in mRNA levels, values were converted logarithmically to calculate geometric means. Parametric test (Student *t* test) or one-way ANOVA test was used to calculate whether differences were statistically significant (*P* < 0.05) using GraphPad Prism software.

### Ethics statement

All animal experiments were performed in BSL2 facilities with protocols that are approved by AALAC-accredited Temple University Lewis Katz School of Medicine Institutional Animal Care and Use Committee (IACUC# 4561) in accordance with guidelines set forth by the USDA and PHS Policy on Humane Care and Use of Laboratory Animals under the guidance of the Office of Laboratory Animal Welfare (OLAW). The institution has an Animal Welfare Assurance on file with the NIH Office for the Protection of Research Risks (OPRR), Number A3594-01.

## Supporting information

S1 Fig Serum from WT and *Stat2^-/-^* mice infected with S. Typhimurium were collected. The concentrations of serum TNFα, IL-6, IL-12 and IL-10 were determined by the BD Cytometric Bead Array (CBA) TH1/TH2 kit according to the manufacturer’s protocol (BD Biosciences). The concentrations of sera cytokines were quantified using a LSRII flow cytometer and analyzed using FloJo software.
(TIF)

S2 Fig Histopathology. Cecal tissue and colon were harvested immediately following euthanasia and fixed in 10% buffered formalin. Tissue was processed according to standard procedures for paraffin embedding, sectioned at 5 μm, and stained with hematoxylin and eosin. A pathologist performed a blinded scoring for inflammatory changes on a rating scale from 0 (not detected) to 5 (severe) for each of the following histological parameters: neutrophil infiltration, submucosal edema, goblet cells, epithelial integrity as described previously [36].
(TIF)

S3 Fig **A.** Neutrophils (PMN) were enumerated in ten fields in H&E stained cecal sections from infected C57BL/6 and *Stat2*^*-/-*^ mice by a blinded pathologist. Numbers were averaged. **B.** Colons of WT (top panel) and *Stat2*^*-/-*^ (bottom panel) uninfected mice were collected and infection and paraffin embedded. Tissues were stained for hypoxia using the pimonidazole hypoxia probe (red) and DAPI (blue). Images were captured at 63x using Leica confocal microscope.
(TIF)

S4 Fig 16srRNA profiling of the fecal microbiome from C57LBL/6 and *Stat2^-/-^* non-cohoused mice. **A.** Major taxa identified through 16s rRNA profiling. **B.** Percent population of Bacteroidetes isolated from C57BL/6 *S*. Typhimurium infected, C57BL/6 uninfected control, *Stat2*^-/-^ *S*. Typhimurium infected and *Stat2*^-/-^ uninfected control mice. **C.** Percent population of Proteobacteria isolated from C57BL/6 *S*. Typhimurium infected, C57BL/6 uninfected control, *Stat2*^-/-^ *S*. Typhimurium infected and *Stat2*^-/-^ uninfected control mice. **E.** CFU/g of *S*. Typhimurium isolated from the feces of C57BL/6 and *Stat2*^-/-^ mice 48 hours post infection with 10^9^ *S*. Typhimurium orally post streptomycin treatment. Mean and SE were calculated by averaging results *p <0.05, as determined by Students t-test.
(TIF)

S5 Fig 16 rRNA profiling of cecal microbiome of C57BL/6 and *Stat2^-/-^* cohoused mice at the genus level. (TIF)

S6 Fig Flow cytometry gating strategy for neutrophils. Neutrophils were gated after duplet and dead cell elimination for the following markers; CD45^+^, Lineage–(CD3^-^, CD56^-^, CD19^-^), Ly6G^+^.
(TIF)
