# Supplementary figures and images for: STAT2 dependent Type I Interferon response promotes dysbiosis and luminal expansion of the enteric pathogen Salmonella Typhimurium
Source: PLoS Pathog. 2019 Apr 22;15(4):e1007745. doi: 10.1371/journal.ppat.1007745 (PMC6513112; doi:10.1371/journal.ppat.1007745)

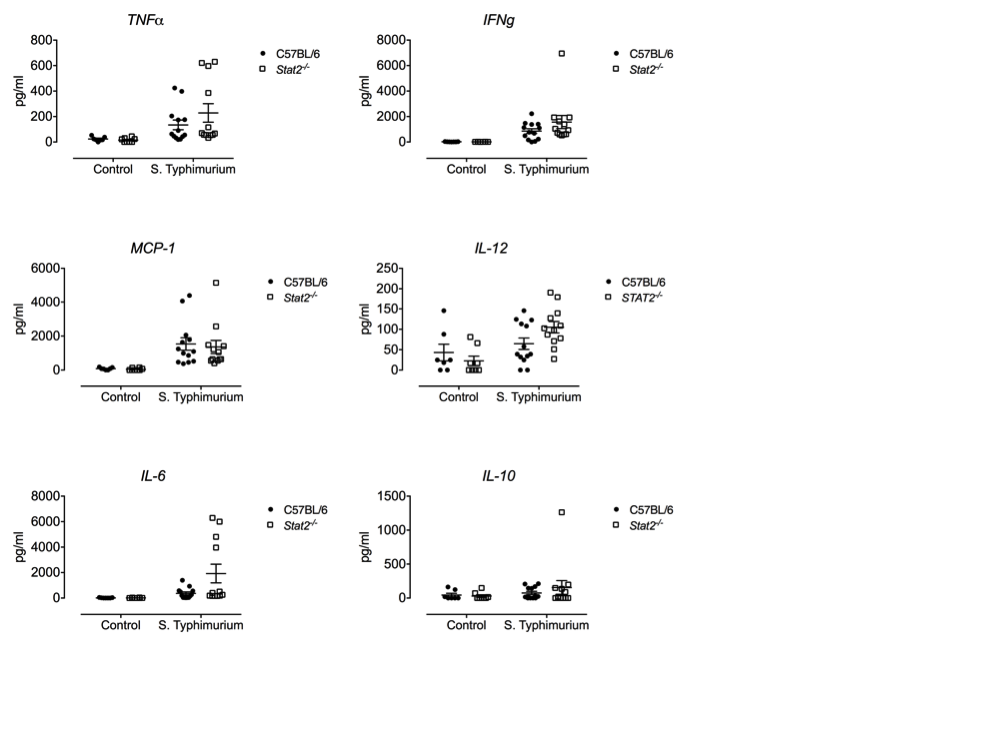

Supplement: S1 Fig — The concentrations of serum TNFα, IL-6, IL-12 and IL-10 were determined by the BD Cytometric Bead Array (CBA) TH1/TH2 kit according to the manufacturer’s protocol (BD Biosciences). The concentrations of sera cytokines were quantified using a LSRII flow cytometer and analyzed using FloJo software. (TIF) [file ppat.1007745.s001.tif]

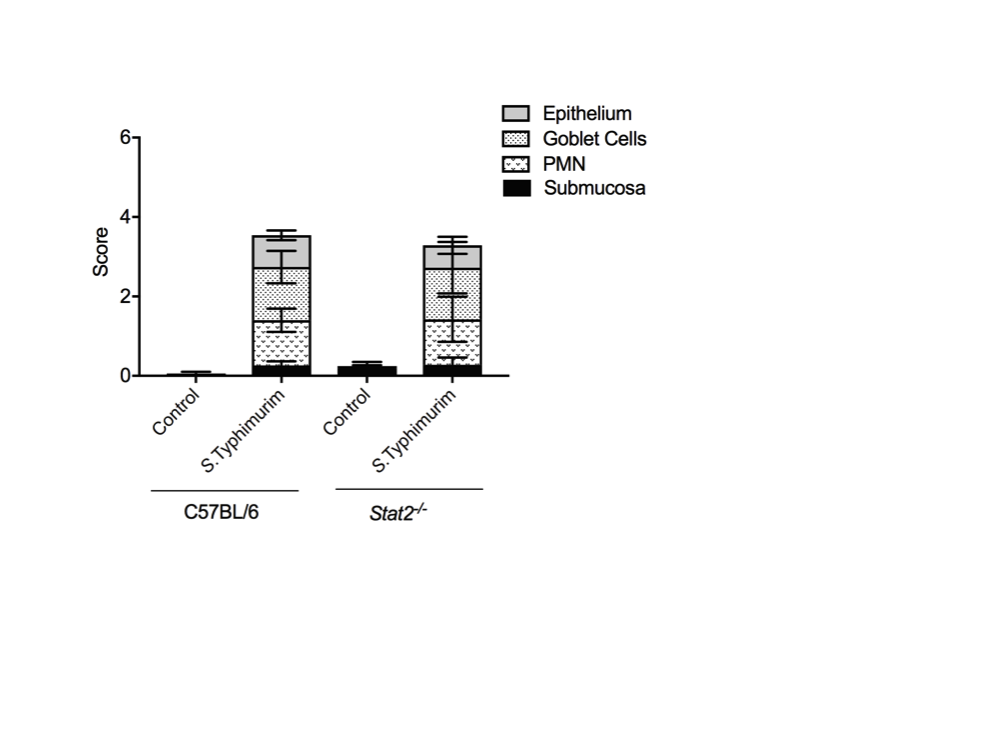

Supplement: S2 Fig — Cecal tissue and colon were harvested immediately following euthanasia and fixed in 10% buffered formalin. Tissue was processed according to standard procedures for paraffin embedding, sectioned at 5 μm, and stained with hematoxylin and eosin. A pathologist performed a blinded scoring for inflammatory changes on a rating scale from 0 (not detected) to 5 (severe) for each of the following histological parameters: neutrophil infiltration, submucosal edema, goblet cells, epithelial integrity as described previously [36]. (TIF) [file ppat.1007745.s002.tif]

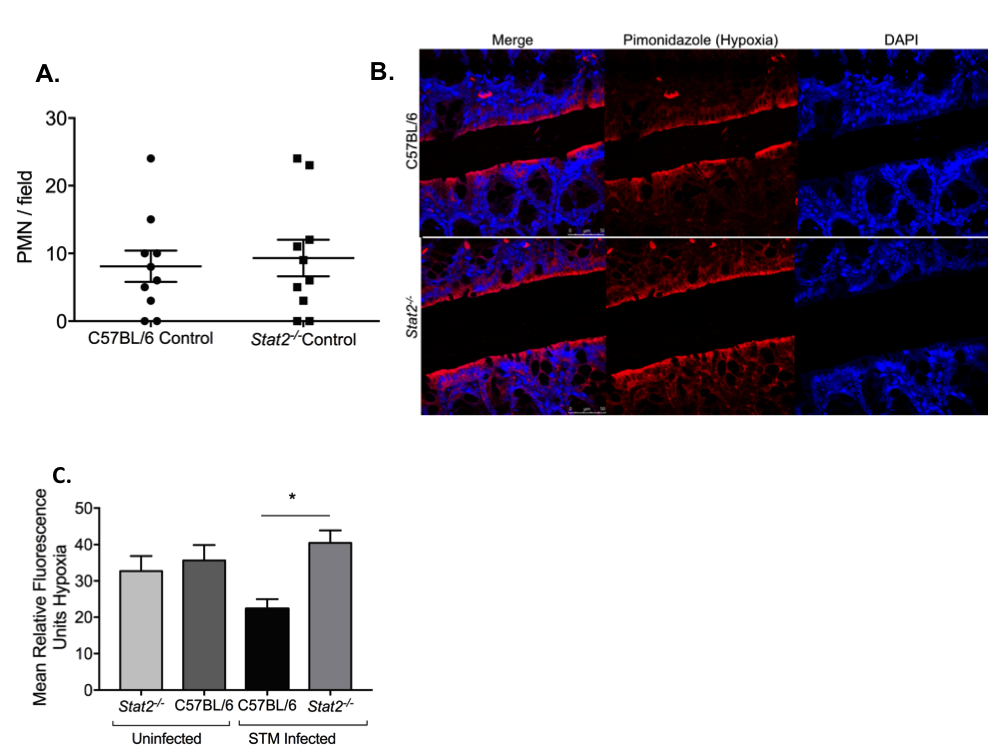

Supplement: S3 Fig — A. Neutrophils (PMN) were enumerated in ten fields in H&E stained cecal sections from infected C57BL/6 and Stat2-/- mice by a blinded pathologist. Numbers were averaged. B. Colons of WT (top panel) and Stat2-/- (bottom panel) uninfected mice were collected and infection and paraffin embedded. Tissues were stained for hypoxia using the pimonidazole hypoxia probe (red) and DAPI (blue). Images were captured at 63x using Leica confocal microscope. (TIF) [file ppat.1007745.s003.tif]

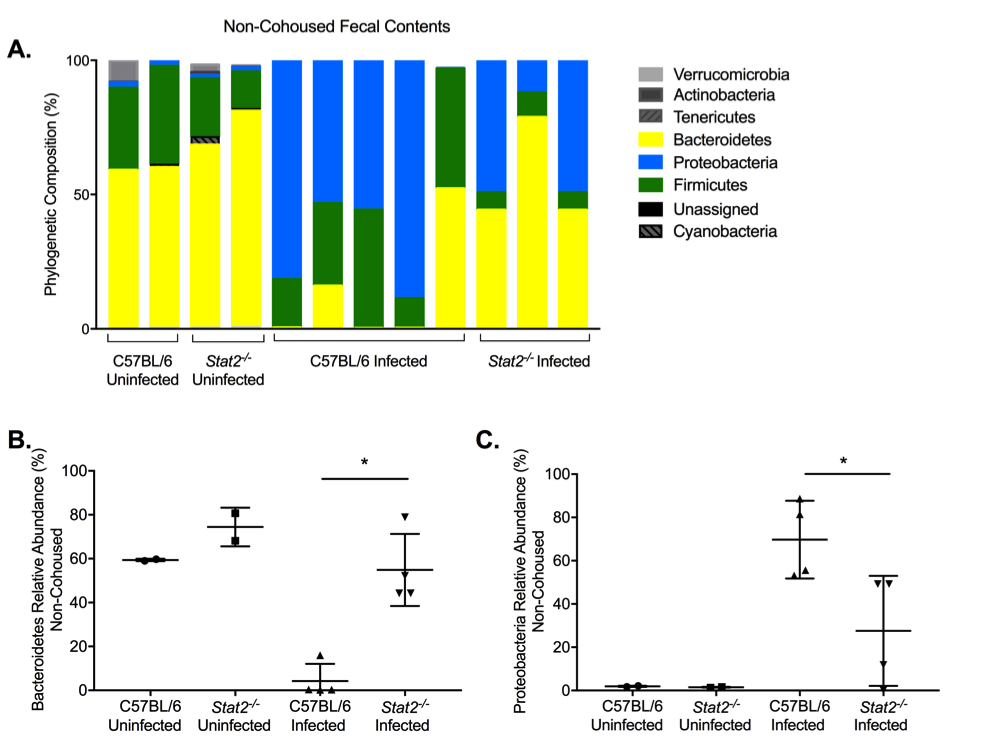

Supplement: S4 Fig — A. Major taxa identified through 16s rRNA profiling. B. Percent population of Bacteroidetes isolated from C57BL/6 S. Typhimurium infected, C57BL/6 uninfected control, Stat2-/- S. Typhimurium infected and Stat2-/- uninfected control mice. C. Percent population of Proteobacteria isolated from C57BL/6 S. Typhimurium infected, C57BL/6 uninfected control, Stat2-/- S. Typhimurium infected and Stat2-/- uninfected control mice. E. CFU/g of S. Typhimurium isolated from the feces of C57BL/6 and Stat2-/- mice 48 hours post infection with 109 S. Typhimurium orally post streptomycin treatment. Mean and SE were calculated by averaging results *p <0.05, as determined by Students t-test. (TIF) [file ppat.1007745.s004.tif]

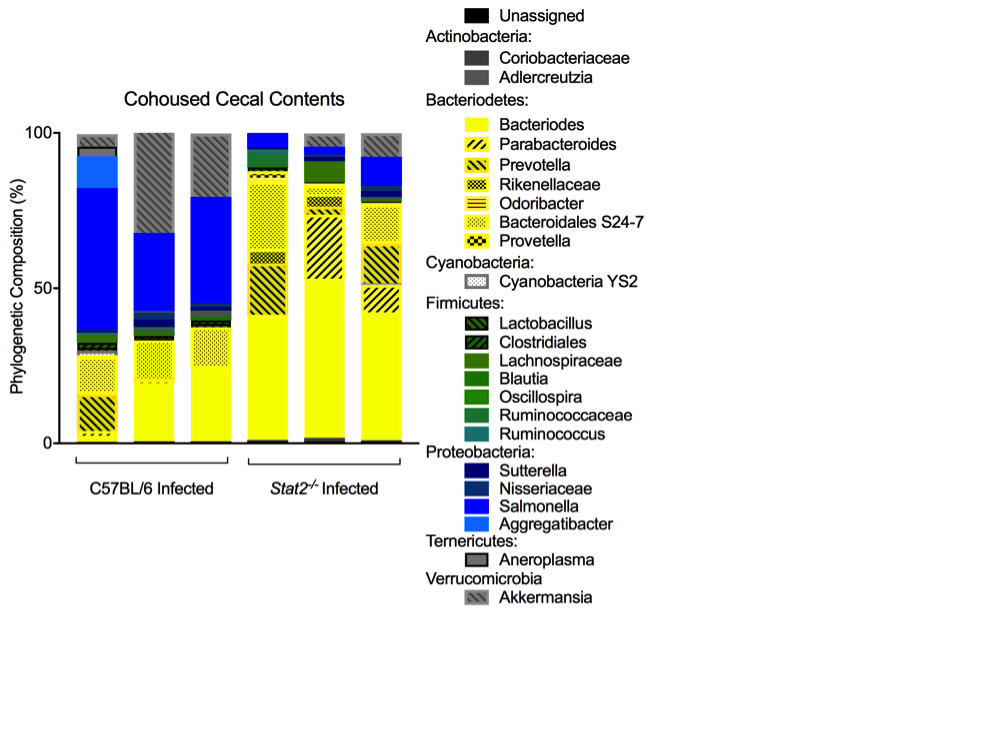

Supplement: S5 Fig — (TIF) [file ppat.1007745.s005.tif]

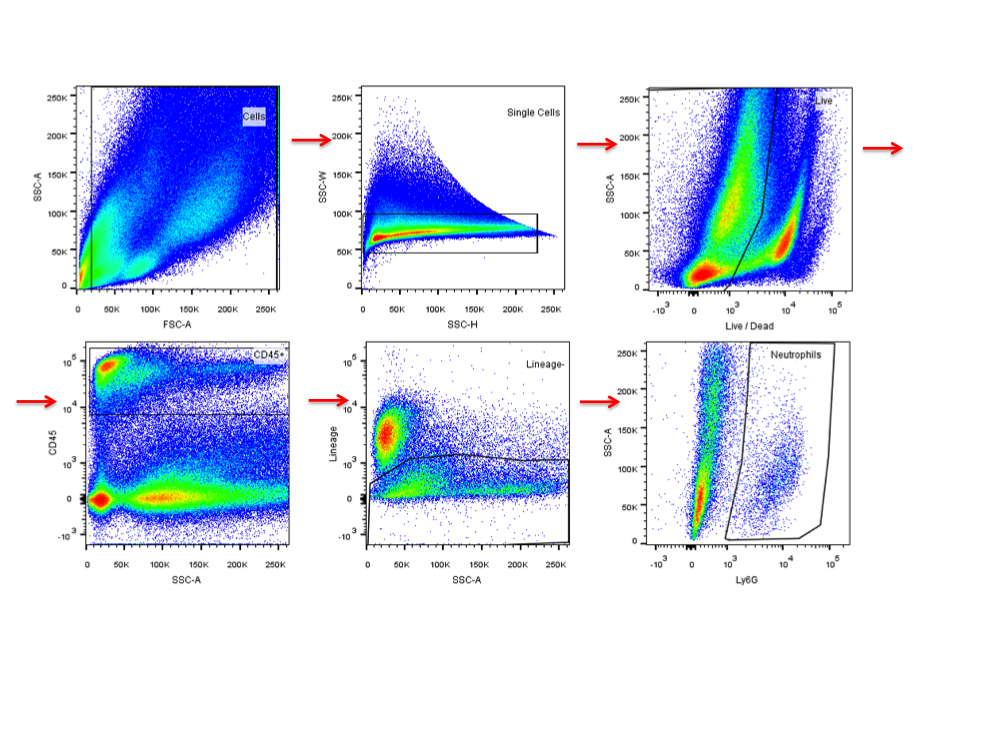

Supplement: S6 Fig — Neutrophils were gated after duplet and dead cell elimination for the following markers; CD45+, Lineage–(CD3-, CD56-, CD19-), Ly6G+. (TIF) [file ppat.1007745.s006.tif]
